# ^18^F-FDG PET/CT Imaging of Metastatic Testicular Choriocarcinoma Mimicking Gastric Cancer which Initial Symptom is Melena

**DOI:** 10.4274/mirt.galenos.2020.65668

**Published:** 2021-02-09

**Authors:** Sibel Göksel, Serkan Akın, Remzi Adnan Akdoğan, Sema Rakıcı, Göksu Yavuz Abdioğlu, Muhammet Ali Ayvaz

**Affiliations:** 1 Recep Tayyip Erdoğan University Faculty of Medicine, Department of Nuclear Medicine, Rize, Turkey; 2 Recep Tayyip Erdoğan University Faculty of Medicine, Department of Medical Oncology, Rize, Turkey; 3 Recep Tayyip Erdoğan University Faculty of Medicine, Department of Gastroenterology, Rize, Turkey; 4 Recep Tayyip Erdoğan University Faculty of Medicine, Department of Radiation Oncology, Rize, Turkey; 5 Recep Tayyip Erdoğan University Faculty of Medicine, Department of Pathology, Rize, Turkey

**Keywords:** Gastric metastasis, melena, testicular choriocarcinoma, 18F-FDG PET/CT

## Abstract

Gastric metastasis of choriocarcinoma is rarely reported in the literature. This case report presents the case of multiple metastatic testicular choriocarcinoma mimicking gastric cancer, with melena as the initial symptom. In this case, ^18^fluorine-fluorodeoxyglucose positron emission tomography/computed tomography (PET/CT) showed that the testis was the primary focus. The contribution of PET/CT is significant to primary focus detection in metastatic diseases of unknown primary origin that presented gastrointestinal bleeding. In addition to its use in staging of testicular carcinoma, PET/CT provides significant benefit in evaluating patients with increased levels of tumor markers and in detecting recurrence.

## Abstract (tr)

Koryokarsinomun mide metastazı literatürde oldukça nadir bildirilmiştir. Bu olgu raporu, mide kanserini taklit eden, başlangıç semptomu melena olan multipl metastatik testis koryokarsinomlu bir hastayı sunmaktadır. Bu olguda, ^18^flor-florodeoksiglukoz pozitron emisyon tomografisi/bilgisayarlı tomografi (PET/BT) testisin primer odak olduğunu gösterdi. PET/BT’nin katkısı, gastrointestinal kanama ile gelen primeri bilinmeyen metastatik hastalıkta primer odak tespitinde önemlidir. Testis kansererinde PET/BT, hastalık evrelendirilmesine sağladığı faydalara ek olarak, özellikle tümör belirteçleri artmış ve nüks hastalık açısından şüpheli olan hastalarda rekürrensi tespit etmede önemli fayda sağlar.

## Figures and Tables

**Figure 1:**
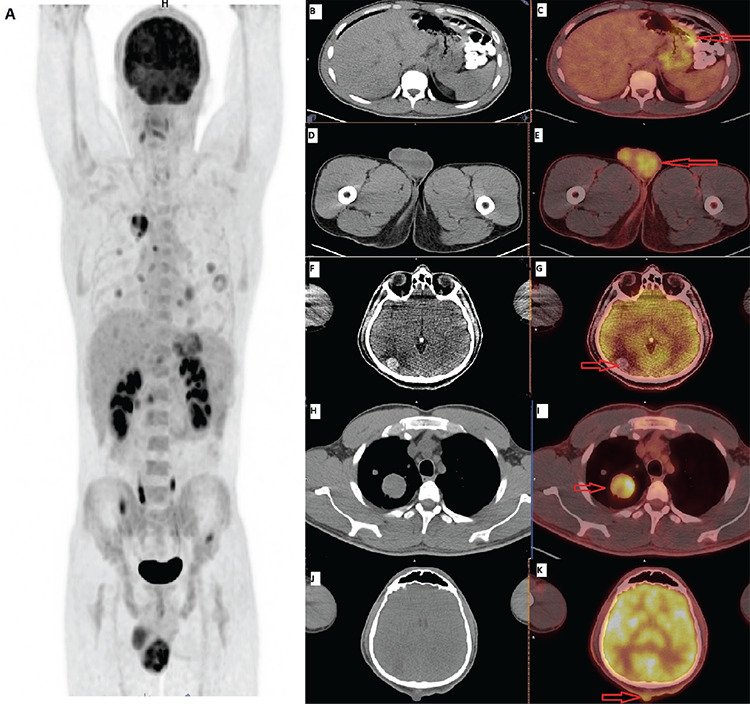
Testicular choriocarcinoma diagnosed with gastric metastases is extremely rare in the literature (1,2,3,4). A 27-year-old male presented with anemia and melena. Polypoid ulcerated lesion on the gastric greater curvature with active bleeding was detected using gastroscopy. The patient underwent ^18^fluorine-fluorodeoxyglucose (^18^F-FDG) positron emission tomography/computed tomography (PET/CT) for clinically suspected gastric cancer. Focal ^18^F-FDG uptake was found on the gastric greater curvature (A, B, C). Metastatic gastrointestinal involvement may be seen in approximately 5% of these cases (5,6). Hypermetabolic focus and asymmetric growth were also found in the left testicle (D, E), and multiple metastatic disease that involves the brain (F, G), lungs (H, I), skin (J, K), liver, lymph node, and bone was detected on PET/CT. Based on PET/CT, all metastases were thought to arise from the testicles. As in this case, in addition to the contribution of PET/CT in diagnosis of testicular cancer, it is very important imaging technique in clinical practice in staging and detection of recurrence (7).

**Figure 2:**
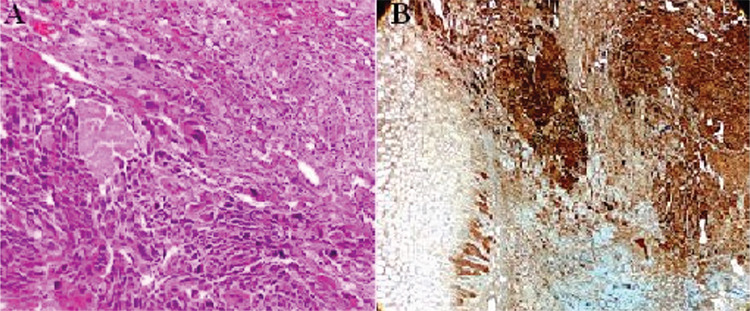
On immunohistochemical examination of the specimen,metastasis of testicular choriocarcinoma was detected in the gastric biopsy specimen. Pathological images of gastric biopsy material. A) Hypercromatic multinuclear and syncytiotrophoblastic cells with large eosinophilic cytoplasm (hematoxylin eosin staining, x400). B) Human chorionic gonadotropin immunohistochemical staining (x400). The germ cell malignancy in young men can present with melena, and malignancy should be suspected in patients presenting with these symptoms.
